# Expression of human α_1_-proteinase inhibitor in *Aspergillus niger*

**DOI:** 10.1186/1475-2859-6-34

**Published:** 2007-10-29

**Authors:** Elena Karnaukhova, Yakir Ophir, Loc Trinh, Nimish Dalal, Peter J Punt, Basil Golding, Joseph Shiloach

**Affiliations:** 1 Center for Biologics Evaluation and Research, Food and Drug Administration, Bethesda,Maryland, 20892 USA; 2 National Institute of Diabetes and Digestive and Kidney Diseases, Bethesda, Maryland, 20892 USA; 3 Department of Microbiology, TNO Quality of Life, 3704 HE Zeist, the Netherlands

## Abstract

**Background:**

Human α_1_-proteinase inhibitor (α_1_-PI), also known as antitrypsin, is the most abundant serine protease inhibitor (serpin) in plasma. Its deficiency is associated with development of progressive, ultimately fatal emphysema. Currently in the United States, α_1_-PI is available for replacement therapy as an FDA licensed plasma-derived (pd) product. However, the plasma source itself is limited; moreover, even with efficient viral inactivation steps used in manufacture of plasma products, the risk of contamination from emerging viruses may still exist. Therefore, recombinant α_1_-PI (r-α_1_-PI) could provide an attractive alternative. Although r-α_1_-PI has been produced in several hosts, protein stability in vitro and rapid clearance from the circulation have been major issues, primarily due to absent or altered glycosylation.

**Results:**

We have explored the possibility of expressing the gene for human α_1_-PI in the filamentous fungus *Aspergillus niger (A. niger)*, a system reported to be capable of providing more "mammalian-like" glycosylation patterns to secretable proteins than commonly used yeast hosts. Our expression strategy was based on fusion of α_1_-PI with a strongly expressed, secreted leader protein (glucoamylase G2), separated by dibasic processing site (N-V-I-S-K-R) that provides *in vivo *cleavage. SDS-PAGE, Western blot, ELISA, and α_1_-PI activity assays enabled us to select the transformant(s) secreting a biologically active glycosylated r-α_1_-PI with yields of up to 12 mg/L. Matrix-assisted laser desorption/ionization mass spectrometry (MALDI-MS) analysis further confirmed that molecular mass of the r-α_1_-PI was similar to that of the pd-α_1_-PI. *In vitro *stability of the r-α_1_-PI from *A. niger *was tested in comparison with pd-α_1_-PI reference and non-glycosylated human r-α_1_-PI from *E. coli*.

**Conclusion:**

We examined the suitability of the filamentous fungus *A. niger *for the expression of the human gene for α_1_-PI, a medium size glycoprotein of high therapeutic value. The heterologous expression of the human gene for α_1_-PI in *A. niger *was successfully achieved to produce the secreted mature human r-α_1_-PI in *A. niger *as a biologically active glycosylated protein with improved stability and with yields of up to 12 mg/L in shake-flask growth.

## Background

Human α_1_-PI is a well-characterized serpin (for recent reviews see [1-4]). Its best known physiological role is the inhibition of neutrophil elastase in the lungs. α_1_-PI is an abundant protease inhibitor in human plasma with a concentration range from 1.04 to 2.76 g/L in healthy individuals and with a half-life of 4–5 days in circulation [5-7]. As a result of a single mutation, α_1_-PI (Z-form) undergoes polymerization and accumulates in the liver, causing a deficiency of α_1_-PI in the blood that may result in progressive, ultimately fatal, emphysema [7].

α_1_-PI is a ~51 kDa single-chain glycoprotein (394 amino acid residues, 12% carbohydrates). It has a typical serpin secondary structure, featuring 9 α-helices, 3 β-sheets and a reactive center loop that is exposed for interaction with a target protease (e.g., review [3]).

Human pd-α_1_-PI is an FDA licensed product, used for replacement therapy in patients with hereditary α_1_-PI deficiency. However, the plasma source itself is limited; moreover, even with testing source material for relevant pathogenic viruses and robust viral clearance steps in the manufacturing process of plasma products, a risk from emerging and yet unknown viruses still remains. As an alternative, and in addition to plasma-derived products, the recombinant versions of α_1_-PI have been under intensive investigation. Since the early 1980s, the human gene for α_1_-PI has been expressed in various hosts, including *E. coli*, yeasts, insect cells, CHO cells, as well as in transgenic plants and animals (see recent review [8]).

We consider the filamentous fungi as a very attractive host for production of human α_1_-PI and other proteins of bio-medical interest. The filamentous fungi systems offer various post-translational modifications to proteins, including glycosylation with the patterns that are more similar to those of mammals than glycosylation provided by common yeast hosts [9-11]. Although these systems have been used for commercial production of enzymes, very few human genes have been expressed in the filamentous fungi [12,13]. Protein size, glycosylation and metastable inhibitory nature of α_1_-PI represent the challenges in this multi-step work in view of an exclusive therapeutic importance of this inhibitor. Moreover, it is worthwhile to mention that multiple efforts of more than 20 years development still did not bring any recombinant α_1_-PI product to the market [8].

The most efficient strategy for expression of mammalian heterologous genes in fungi is a production of the target protein as a fusion protein linked to the C-terminus of a highly expressed and well secreted native fungal protein [14-16]. To release the target protein from the fusion chimera, the *in vivo *cleavage is accomplished by introducing the KEX2-type protease recognition site at the fusion junction [17-20].

In the present study we examined the suitability of filamentous fungus *A. niger *for the expression of the human gene for α_1_-PI. We successfully achieved heterologous expression of the gene for α_1_-PI in *A. niger *to produce the secreted mature human r-α_1_-PI as a biologically active glycosylated protein with improved stability and with yields of up to 12 mg/L in shake-flask growth.

## Results

### 1. Expression vector, transformation and selection

**Figure 1:**
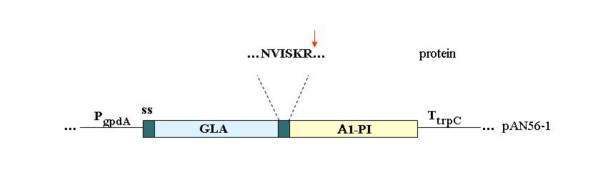
Diagram of the fusion region between glucoamylase (GLA) and α_1_-PI coding region (A1-PI) within the pAN56-1 expression vector showing the KEX2 cleavage sequence (see abbreviations in the text).

The expression cassette of pAN56-1/α_1_-PI (Fig. 1) was constructed as described in Methods. The correct insertion of α_1_-PI was verified by restriction digest and DNA sequence analysis. Co-transformation of the protoplasts of *A. niger *strain D15#26 with pAN56-1/α_1_-PI and pBLUE-AmdSPyrG (further referred as PyrG), followed by the selection on uridine-deficient media enabled us to isolate the transformants containing the selection fragment. Further screening for α_1_-PI production was performed by direct detection of the secreted target protein in the supernatants of shake-flask cultures.

### 2. Evaluation of proteolytic digestion by fungal proteases

Initially, the *A. niger *strain AB4-1 was used as the parental host strain for transformation. However, during the screening for r-α_1_-PI in shake-flask cultures we were unable to detect the target protein in the supernatants from the selected transformants. No band was seen on Western blots at the electrophoretic mobility of the standard pd-α_1_-PI, thus suggesting that either the transformants contained only the PyrG plasmid, but not the expression vector, or that the target protein is not visible because of proteolysis with native fungal proteases. This prompted us to evaluate the possibility that α_1_-PI might be proteolytically digested under the conditions of shake-flask growth. Given this reasoning, a protease-deficient host strain, D15#26, was chosen that resulted in successfully produced amounts of r-α_1_-PI. (However, several other effects, such as the efficiency of transcription from the sites of random integration in different transformants, or differences in the gene copy number between strains (not pursued in this study) may also result in significant differences in expression levels.)

**Figure 2:**
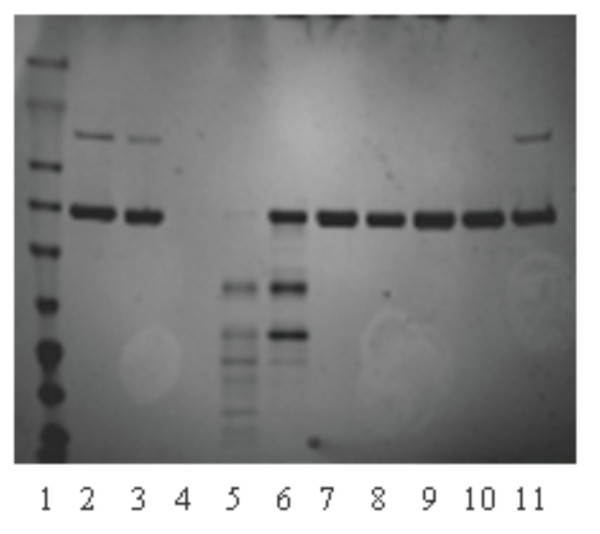
SDS-PAGE analyses of proteolytic digestion. 1 – protein ladder; 2 and 11 - α_1_-PI standard; 3 - α_1_-PI standard kept O/N at RT; 4 – Supernatant (S) from 5 days growth of *A. niger *AB4-1; 5 - α_1_-PI + S (initial, pH 3.5) O/N, RT; 6 - α_1_-PI + S (initial, pH 3.5) O/N, 4°C; 7 - α_1_-PI + S pH 7.3 kept O/N at RT; 8 - α_1_-PI + S pH 7.3 kept O/N at 4°C; 9 - α_1_-PI + S pH 8.4 kept O/N at RT; 10 - α_1_-PI + S pH 8.4 kept O/N at 4°C. The initial concentration of α_1_-PI standard in all samples was 5.175 mg/mL.

Fig. 2 shows the results of the basic experiment conducted with standard pd-α_1_-PI, which was spiked with supernatant from 5 days growth of parental *A. niger *strain AB4-1 transformed with PyrG plasmid only. Whereas standard α_1_-PI in Tris buffer, pH 8.4 used as a control, was stable during the time of the experiment (10 h) both at 4°C and RT, the same amounts of α_1_-PI spiked with supernatant of *A. niger *growth (naturally acidified during growth to pH of ~3.5–4.5) showed quick decay due to proteolysis that proceeded significantly faster at room temperature (RT).

Two strategies were employed to minimize proteolysis in this system. Proteolytic activity of native fungal proteases is mainly neutralized by keeping the pH above 7. Secondly, a strain of *A. niger *more deficient in proteases, non-acidifying mutant D15#26 was used (instead of AB4-1).

### 3. Analyses of the expression of α_1_-PI in *A. niger*

**Figure 3:**
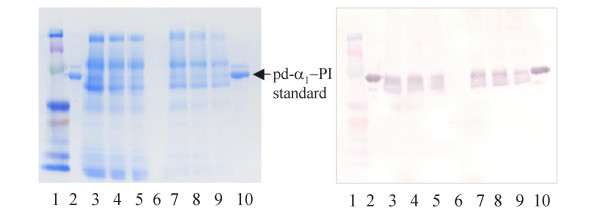
SDS-PAGE and Western blot (right panel) analysis of α_1_-PI expression in *A. niger *D15#26. 1-protein ladder, 2 and 10 - pd-α_1_-PI standard, 3–5 - supernatant from growth of the transformant #1 (30, 20, and 10 μL respectively), 6 – supernatant from growth of PYRG-transformant, 7–9 - supernatant from growth of the transformatant #2 (the 30, 20, and 10 μL respectively).

The results reported here utilize the D15#26 strain and pH above 7.0, as indicated above. The supernatant samples from the cultures were assayed by ELISA. In addition, to minimize the possibility of false interpretation of the ELISA results, SDS-PAGE and Western blot analysis were used, since it was shown by us that the ELISA can detect peptides derived from digestion of α_1_-PI [21]. Fig. 3 shows a typical SDS-PAGE and complimentary Western blot for the supernatant of the selected D15#26 transformant taken after 96 hours of growth (lanes 3–5). Lanes 6–9 corresponds to another D15#26 selected transformant. The yields estimated by ELISA and by Western blot were in agreement indicating secretion of up to 12 mg/L of the target protein. The r-α_1_-PI was shown to be functionally active (see below). No cytoplasmic accumulation was detected in the control analysis of the *A. niger *cell extract.

### 4. Characterization of r-α_1_-PI from *A. niger*

**Figure 4:**
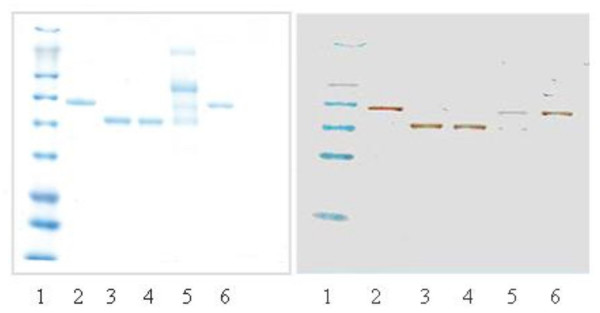
SDS-PAGE and Western blot (right panel) comparison of different α_1_-PI's: 1- protein ladder; 2 and 6 - pd-α_1_-PI standard; 3 - deglycosylated pd-α_1_-PI; 4 - α_1_-PI from *E. coli *(eluted from TALON beads); 5 - r-α_1_-PI in the supernatant from *A. niger *D15#26.

Fig. 4 compares r-α_1_-PI from *A. niger *with standard pd-α_1_-PI, enzymatically deglycosylated pd-α_1_-PI (de-pd-α_1_-PI), and with r-α_1_-PI from *E. coli*. Unlike r-α_1_-PI in the soluble protein fraction from *E. coli *(see SDS-PAGE in [21]), the raw supernatant from *A. niger *has a relatively simple protein composition.

### 4.1. HPLC analyses

**Figure 5:**
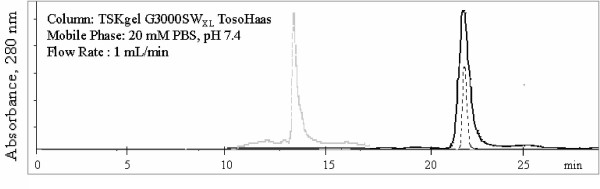
Evaluation of stability of r-α_1_-PI from *A. niger *(solid trace) and from *E. coli *(dashed trace) by SE-HPLC. The fractions of r-α_1_-PI were collected by HPLC, kept on ice and re-injected. The gray dashed trace reflects polymerization of r-α_1_-PI from *E. coli *as shown 1.5 h later after elution, while no aggregation was observed for r-α_1_-PI from *A. niger *for ~12 hours; all analytes were stored at 4°C.

Size-exclusion (SE) HPLC data was used for the stability evaluation of the r-α_1_-PI's. The fractions corresponding to the α_1_-PI peak with a retention time of 21 min were collected and stored on ice. It is noteworthy that the retention times of r-α_1_-PI's from *A. niger *and from *E. coli *are essentially the same as that of pd-α_1_-PI (~21 minutes in the conditions used). Fig. 5 demonstrates that whereas the non-glycosylated r-α_1_-PI from *E. coli *(dashed trace) undergoes rapid aggregation (as observed by accumulation of the peak with a retention time of ~13 min corresponding to polymerized α_1_-PI, gray trace), the glycosylated r-α_1_-PI from the *A. niger *supernatant (solid trace) is relatively stable during at least 12 hours.

### 4.2. Evaluation of molecular mass

The molecular mass sizes of r-α_1_-PI's were evaluated by SDS-PAGE and Western blot analysis, using pd-α_1_-PI and its deglycosylated version as the references (Fig. 4). In addition, we performed mass-spectrometric analysis of the proteins using MALDI-MS in the conditions established earlier [22]. α_1_-PI is a heterogeneous protein due to intrinsic carbohydrate diversity and some differences in the polypeptide part [23-26]. Therefore, in the MALDI-MS spectra the molecular ion and the related ions are represented by its ion distribution clusters, and the molecular mass values are assigned by the major ion peak at which the cluster is centered. The molecular weight of the standard pd-α_1_-PI (spectra not shown) is determined by the observed molecular ion [M+H]^+ ^at 50,300 Da (Table 1), which is in agreement with its half-mass ion [M/2+H]^+ ^detected at 25,150 Da. Mass spectrum of r-α_1_-PI from *A. niger *shows the molecular ion cluster centered at 50,130 Da, which is close to that of plasma α_1_-PI standard. Therefore, these results allow for more accurate molecular mass values and suggest that there is no "hyperglycosylation" in case of r-α_1_-PI secreted from *A. niger*. Enzymatically deglycosylated pd-α_1_-PI showed the main molecular ion at 44,210 Da, therefore, serving as an additional non-glycosylated reference.

**Table 1:** Activity of r-α_1_-PI from *E. coli *and *A. niger *and evaluation of molecular weight of recombinant and plasma-derived species determined by MALDI-MS

Protein	Activity^a ^(%)	Molecular mass^b^
pd-α_1_-PI (standard)	100	50,300
degly-pd-α_1_-PI	n.a.^c^	44,210
r-α_1_-PI/*E. coli*	35^d^	45,000^e^
r-α_1_-PI/*A. niger*	76^f,g^	50,100

^a^The calculations were based on comparison with pdtα_x_tPI as a standard (100%) and using normalized equal concentrations of r-α_1_-PI samples as determined by ELISA; ^b^molecular weight is shown as a an average value measured for 2 samples; ^c ^not available; pd-α_1_-PI was enzymatically deglycosylated under denaturing conditions; ^d ^as measured for His-tagged r-α_1_-PI within 1.5 h after elution from the TALON beads; ^e ^as estimated by amino acid sequence for α_1_-PI without glycans; ^f ^shown for *A. niger *supernatant after Amicon 10 K filtration procedure; ^g ^the standard deviation of our potency assay is ± 15%.

### 4.3. Activity of r-α_1_-PI

Inhibitory activity of the recombinant α_1_-PI secreted into the supernatant was evaluated against porcine pancreatic trypsin, using pd-α_1_-PI as a standard (assigned as 100%, Table 1). The α_1_-PI assay samples were adjusted to the same initial concentration and subjected to the same dilutions on the plate. Basal (low) response of the supernatant from the growth of D15#26 transformed with PyrG only was subtracted. Activity of the r-α_1_-PI from *A. niger *was not less than 75% of the standard (*e.g*., to inhibit 0.7 μmole active PPT about 0.92 μmole of r-α_1_-PI was required). This activity was significantly higher than that of r-α_1_-PI from *E. coli *(~35%). The latter correlates well with the fact that non-glycosylated r-α_1_-PI from *E. coli *tends to aggregate rapidly with subsequent loss of activity.

## Discussion

In this paper we demonstrate that it is possibile to express the human gene for α_1_-PI in the filamentous fungus *A. niger *as a secreted glycosylated protein with stability that is significantly improved in comparison with non-glycosylated recombinant protein from *E. coli*. The secreted r-α_1_-PI was characterized in comparison with pd-α_1_-PI and its enzymatically deglycosylated version (de-pd-α_1_-PI) used as the "*in-house*" standards, as well as with non-glycosylated r-α_1_-PI produced in *E. coli *[27].

*A. niger *strains have been already used as hosts for the production of other serine proteinase inhibitors. Mikosch et al. reported on the secretion of active human mucus proteinase inhibitor (antileukoproteinase), which is a 11.7 kDa non-glycosylated single chain protein stabilized by eight disulfide bonds [16]. Later, MacKenzie et al. reported on an aberrant processing of bovine pancreatic trypsin inhibitor (known as aprotinin, a small polypeptide of 58 amino acid residues) secreted by *A. niger *[20]. However, to the best of our knowledge, our work shows for the first time that human α_1_-PI, a complex glycoprotein of medium size (394 amino acid residues, ~50.3 kDa) and of significant therapeutic value, can be successfully produced in this system.

The expression of α_1_-PI in *A. niger *was designed to obtain the recombinant inhibitor in the secreted glycosylated form with enhanced yield. This was successfully achieved by fusion of the α_1_-PI coding sequence downstream of the glucoamylase truncated gene (glaA_G2_), under transcriptional control of the constitutively expressed glyceraldehydes-3-phosphate dehydrogenase (gpdA) promoter, according to the earlier established strategy [14-16]. The efficiency of protein production was evaluated by the level of expression by direct determination of the secreted r-α_1_-PI in the supernatant during growth.

To minimize possible interactions of native fungal proteases with the target recombinant inhibitor during growth, the following changes were implemented: (a) a protease-deficient mutant D15#26 was used for transformation (instead of AB4-1), and (b) the pH of the supernatants was maintained above 7.0. Screening for the target protein was routinely assayed by ELISA, in a protocol recently developed by us for this purpose [21]. However, given the subtle nature of α_1_-PI and the challenge of producing this medium size inhibitor in its biologically active form, standard SDS-PAGE/Western blot analysis was also conducted to visualize evidence for degradation peptides. As an analytical tool, ELISA was utilized to quantify r-α_1_-PI's production under various growth conditions. Maximum yields of r-α_1_-PI achieved in shake-flask cultures were at 12 mg/L, after 96 hours of batch culture growth, which is comparable with those reported for other mammalian proteins (10 mg/L) that were obtained in *Aspergillus *strains [16,17,28]. Although these yields of r-α_1_-PI appear to be lower than the yields we achieved in *E. coli *(20 mg/L and 38 mg/L in raw extracts before purification), the protein obtained from *A. niger *is significantly more stable than the non-glycosylated α_1_-PI versions from *E. coli*, as evaluated by SE-HPLC.

Furthermore, inhibitory activity of r-α_1_-PI from *A. niger *is significantly higher than that of non-glycosylated r-α_1_-PI version from *E. coli*, which tends to aggregate more rapidly, thereby losing its inhibitory activity. This is consistent with the previously reported data on activity and stability of r-α_1_-PI's that were produced in other host systems [8], thus confirming that low stability results in lower potency.

As was reported earlier for bovine pancreatic trypsin inhibitor [20], the possibility for aberrant processing of the fusion protein by KEX2-like endoprotease may result in a mixture of target proteins differing at the N-terminus. Although this possibility was not evaluated for r-α_1_-PI obtained from shake-flask cultures, the secreted r-α_1_-PI had high inhibitory activity (not less than 75%) in this system, suggesting that r-α_1_-PI was mostly processed correctly. Although a higher yield was mentioned for r-α_1_-PI produced in a fermentor [8], it related to a total r-α_1_-PI that contained certain amounts of latent (inactive) and digested α_1_-PI species. The optimization for a semi-large scale production of r-α_1_-PI in a fermentor with all parameters controlled is currently under development [29].

As the secreted protein, r-α_1_-PI is glycosylated, and the SDS-PAGE and Western blot demonstrate that the electrophoretic mobility of r-α_1_-PI from *A. niger *is comparable with that of pd-α_1_-PI standard. Together with the activity results and SE-HPLC data, it suggests a correct cleavage by KEX2-like site and an appropriate folding of the secreted protein. MALDI-MS data provide additional proof that the average molecular mass of the recombinant protein (~50,100 Da) is close to that observed for pd-α_1_-PI (50,300 Da), and therefore, the sizes of glycans in both are comparable. Although the results of testing with PNGase F suggest that glycosylation is predominantly of N-type, more detailed glycan characterization could be of interest in view of the comprehensive glyco-proteomic analysis recently performed by Kolarich et al. [25,26] for native human α_1_-PI.

## Conclusion

As a part of our multi-step investigation of α_1_-PI, we have successfully expressed the human gene for α_1_-PI in the filamentous fungus *Aspergillus niger*, as a fusion protein with glucoamylase G2, a strongly expressed secreted leader protein, separated by a processing peptide sequence to allow *in vivo *cleavage. SDS-PAGE, Western blot, ELISA and inhibitory activity assays enabled us to select the transformant(s) that were capable of secreting biologically active glycosylated r-α_1_-PI with improved stability and with yields of up to 12 mg/L. MALDI-MS analysis further confirmed that molecular mass of the r-α_1_-PI was similar to that of native plasma protein, thus suggesting that there was no "hyperglycosylation" from the host. Taken together, the results of our shake-flask experiments suggest the feasibility of this system for further development of r-α_1_-PI, a protein of our particular therapeutic interest.

## Methods

### 1. α_1_-PI references, reagents and solutions

α_1_-PI from CalBiochem (Darmstadt, Germany) was used as an "in-house" α_1_-PI standard which was qualified as earlier described [21]. The concentrations of the purified α_1_-PI preparations were determined spectrophotometrically using a coefficient of extinction A_280_^0.1% ^0.433 [30]. Deglycosylated and non-glycosylated α_1_-PI reference samples were prepared as described below. Bovine serum albumin, trypsin from porcine pancrease, *p*-nitrophenyl *p*'-guanidino-benzoate hydrochloride (NPGB), *N*-benzoyl-L-arginine *p*-nitroanalide hydrochloride (BApNA), 2-mercaptoethanol, anhydrous dibasic sodium phosphate, 3,3',5,5'-tetra-methylbenzidine (TMB) liquid substrate system for membrane and TMB for ELISA, isopropyl β-D-1-thiogalacto-pyranoside (IPTG), PNGase F and sinapinic acid were from Sigma Chemical Co. (St. Louis, MO). Hexafluoroisopropanol was from Brand-Nu Laboratories (Meriden, CT). Cellulase was from Interspex Product (San Mateo, CA). Phosphate buffered saline (D-PBS) without Ca & Mg was from Quality Biological, Inc., (Gaithersburg, MD). Simply Blue^TM^ SafeStain and SeeBlue Plus2^®^ Pre-Stained Standard were from Invitrogen (Carlsbad, CA). All other chemicals were ACS reagent grade from Fisher Scientific (Pittsburgh, PA). Antibodies for Western blot: goat anti-human α_1_-PI affinity purified and rabbit anti-goat alkaline phosphatase (AP) conjugate were from Jackson ImmunoResearch Laboratories (West Grove, PA). AP Conjugate Substrate Kit to visualize the alkaline phosphatase in Western blot was from Bio-Rad Laboratories (Hercules, CA). Antibody for ELISA: rabbit anti-human α_1_-PI (capture antibody) from Sigma Chemical Co. (St. Louis, MO) and sheep anti-human α_1_-PI-HRP (antibody-enzyme conjugate) from BioDesign International (Saco, ME).

### 2. Strains

*Aspergillus niger *(pyrG^-^) strains AB4-1 (parental) and D15#26, a protease-deficient, non-acidifying mutant [31] from TNO were used for transformation and production of glycosylated r-α_1_-PI. *E. coli *strain TOP10 used for construction and propagation of vectors was from Invitrogen Co. (Carlsbad, CA). *E. coli *strain JM109 used for cDNA preparation was from Promega Co. (Madison, WI).

### 3. Culture conditions

For selection of the *A. niger *transformants, the following selective solid minimal uridine-deficient medium was used containing (per 1 liter): 5.95 g NaNO_3_, 0.52 g KCl, 1.5 g KH_2_PO_4_, 0.24 g MgSO_4_, 1% (wt/vol) glucose, and trace elements (1,000 × stock: 12.27 g ZnSO_4_, 3.15 g MnCl_2_, 5.0 g FeSO_4_× 7H_2_O,0.92 g CoCl_2_, 1.02 g CuSO_4_, 1.28 gNa_2_MoO_4_, 50.8 g EDTA).

The biomass for protoplasting and transformation was obtained by growing in the complete media that contained (per 1 liter): 6 g NaNO_3_, 0.52 g KCl, 0.68 g KH_2_PO_4_, 1.045 g K_2_HPO_4_, 2.77 g MgSO_4 _× 7 H_2_O, 1 g yeast extract, 1 g casamino acids, 10 g glucose, 1 ml vitamins solution (per 100 ml: 0.01 g pyridoxine-HCl, 0.015 g thiamine-HCl, 0.075 g *p*-aminobenzoic acid, 0.25 g nicotinic acid, 0.25 g riboflavin, 2.0 g choline-HCl, 0.005 g biotin), 1 ml trace solution (per 100 ml: 2.2 g ZnSO_4_, 1.1 g H_3_BO_3_, 0.5 g MnCl_2 _× 4 H_2_O, 0.5 g FeSO_4 _× 7 H_2_O, 0.16 g CoCl_2 _× 6 H_2_O, 0.16 g CuSO_4_, 0.11 g (NH_4_)_2_MoO_4_, 6.5 g EDTA tetrasodium salt) supplemented with 10 mM of uridine.

### 4. DNA and plasmids

The gene for human α_1_-PI was kindly provided by Dr. Sanio Woo (Mount Sinai, NY, NY). The pAN56-1 vector and PyrG selection plasmid were from Dyadic Nederland BV.

### 5. Construction of expression plasmid

The expression vector pAN56-1/α_1_-PI (Figure 1) was constructed using established molecular methods protocols [32]. A full length 1230 bp cDNA encoding the mature human α_1_-PI (GenBank Accession # K01396) was generated by PCR flanked by BstB1 restriction sites and linked with nucleotide sequence encoding a dibasic processing site (N-V-I-S-K-R). The following primers were used: (1) 5' TTCGAATGTGATATCCAAGCGCGGAGATCCCCAGGGAGATGCTGCCC containing a processing sequence (underlined), and (2) 3' TTCGAATTATTTTTGGGTGGGATTCACCACTTTTCCCATGAAGAGGGGTGGG. PCR was performed using Model PTC-200 of Peltier Thermal Cycler (MJ Research, Inc.). The PCR fragment was then ligated into the plasmid pCR2.1 TOPO (Invitrogen Co., Carlsbad, CA) and the sequence of the amplification product was confirmed by sequencing at the CBER FDA core facilities. Excising the fragment by BstB1 allows the subcloning into the NarI digested expression vector pAN56-1 (from TNO, GenBank Accession # Z32700) to generate pAN56-1/α_1_-PI. The cloning resulted in expression cassette that contained the constitutively expressed glyceraldehydes-3-phosphate dehydrogenase promoter (P_gpdA_), gene for mature human α_1_-PI fused to the coding region of glucoamylase truncated gene (GLA) linked by the processing site, followed by the trpC terminator (T_trpC_).

### 6. Transformation and selection of *A. niger* transformants

The *A. niger *strain D15#26 was grown in minimal media (above) for 16 h at 30°C with shaking at 150 rpm. The protoplasts preparation and transformation was followed as described in [33] with the exception of using 5 mg of cellulase per mL of wet mycelia instead of NovoZym 234. The protoplasts were co-transformed with the expression vector pAN56-1/α_1_-PI and PyrG selection plasmid. PyrG transformants were selected on plates of solid minimal media without uridine, prepared with 15 g/L Oxoid agar. The plates were incubated at 30°C for 2 days until fungal colonies became visible. Large colonies were selected and subsequently transferred onto new plates prior to screening for α_1_-PI production in minimal liquid media.

### 7. Screening for α_1_-PI producers by SDS-PAGE and Western blot

The selected PyrG transformants were screened for the appearance of r-α_1_-PI in the supernatants during growth (200 mL per 1000 ml flask at 28°C on rotary shaker at 150 rpm and incubated for 5 days; pH was maintained above 7.0 by using 2 M NaOH solution). The aliquots of the supernatants were collected during the 5 days growth and analyzed for the presence of secreted r-α_1_-PI by SDS-PAGE and Western blot analysis using pre-cast 7.5% and 4–20% Tris/Gly mini-gels under reducing conditions. Simply Blue^TM^ SafeStain was used for staining, and SeeBlue Plus2^®^ Pre-Stained Standard served as the protein ladder. Goat anti-human α_1_-PI affinity purified and rabbit anti-goat alkaline phosphatase (AP) conjugate followed by detection with AP Conjugate Substrate Kit were used in the Western blot to visualize the protein. Based on the screening, the best transformant was selected for further studies.

### 8. Quantification of r-α_1_-PI by ELISA

Quantification of r-α_1_-PI in raw biological samples was performed as in [21]. The samples were assayed *in triplicate*. Spiking with the supernatant aliquots adjusted to pH 7.3 and 8.4 have been conducted similarly. The blank supernatant from growth of *A. niger *transformed with PyrG plasmid only, was used as a matrix to confirm the specificity of the antigenic determination.

### 9. Evaluation of α_1_-PI proteolytic digestion by fungal proteases

To evaluate for possible proteolytic degradation of r-α_1_-PI during growth, the standard pd-α_1_-PI was diluted to 5.175 mg/mL by adding Tris buffer or *A. niger *supernatant from the strain D15#26. The samples (in Tris and in supernatant) were incubated at 4°C and at RT for overnight (O/N), and evaluated by ELISA according to the protocol earlier described [21].

### 10. SE-HPLC

SE-HPLC analysis was carried out on the System Gold^® ^HPLC (Beckmann Corp.) controlled by 32 Karat Work station software. The stationary phase: two TosoHaas TSK-3000SW_XL _columns (5 *μ*m, 7.8 mm × 30 cm) connected in series and an SW_XL _guard column. The mobile phase: PBS buffer, pH 7.4. The flow rate: 1 mL/min. Detection: absorbance at 280 and 215 nm.

### 11. Activity assay

The inhibitory activities of r-α_1_-PI's produced in *A. niger *and in *E*. *coli *were evaluated against trypsin from porcine pancrease. Titration of trypsin active sites was performed using NPGB as an active-site titrant according to the established procedure [34]. Our plate-based version of this assay reproducibly showed 76% of the active sites in the porcine pancreatic trypsin. Determination of the inhibitory activity of α_1_-PI is based on measuring the residual assay protease activity after trypsin interaction with various amounts of α_1_-PI. The inhibitory activity of r-α_1_-PI was determined in comparison with an in-house standard (100%) and using BApNA as a chromogenic substrate. The residual trypsin activity was measured by monitoring the absorbance of the released *p*-nitroanilide at 405 nm (molar extinction coefficient of 10,500 M^-1^cm^-1^).

The samples of r-α_1_-PIs from *A. niger *and *E*. *coli *were prepared for assaying inhibitory activity as follows. (a) r-α_1_-PI from *E. coli*. Soluble cytosolic protein fractions from *E*. *coli *biomass were subjected to purification on TALON beads, the eluted fractions containing r-α_1_-PI were collected and concentrated using Amicon (10 K) filtration at 13,000 rpm; the concentrate was analyzed by SE-HPLC, and the fraction eluted at 21 min was collected and placed on ice. The r-α_1_-PI concentration was determined spectrophoto-metrically, and its activity was immediately assayed. (b) Inhibitory activity of r-α_1_-PI from *A. niger *transformants was evaluated as follows. The supernatants (pH 7.0) were concentrated and desalted by aid of filtration on Amicon 10 K and kept on ice before use the same day. Samples in 1 mL aliquots were mixed with equal volume of Tris buffer, pH 8.4, and cleared by centrifugation at 24,000 rpm for ~5 min. The r-α_1_-PI concentration was determined by ELISA as described above. Trypsin solution in Tris buffer served as a control protease sample. After mixing with the samples containing standard α_1_-PI and r-α_1_-PI samples, the mixtures were incubated for 15 min at RT. After adding 100 μL of the substrate solution, simultaneously by using a multi-channel pipette, the residual trypsin activity was immediately monitored as end point kinetics at 405 nm on THERMOmax™ microplate reader (Molecular Devices Co., Menlo Park, CA).

Trypsin active site titration and the inhibitory assay were performed in duplicate at 25°C in Tris buffer (pH 8.4). Back calculations for activities of r-α_1_-PIs in the original samples were performed using the corresponding dilution factors.

### 12. MALDI-MS

A linear time-of-flight instrument with delayed extraction (Voyager-DE, Applied Biosystems, Framingham, MA) was used. Mass calibration was performed using bovine serum albumin (66,500 Da) as an internal standard. The average of 50–200 laser shots was used for recording the mass spectra within the acquisition mass range of 15,000 – 80,000 Da. The samples were prepared as described in [22] with minor changes as following. The samples (1 μL of supernatants containing α_1_-PI or standard pd-α_1_-PI) were loaded onto a gold-plated sample plate and allowed to air dry. The matrix solution was prepared by mixing 12 μg of sinapinic acid with 300 μL of 0.1% trifluoroacetic acid/acetonitrile (1:1, v/v). After spinning down at 5,000 rpm, 1 μL of the resulting solution was loaded on the top of each spot of the sample and allowed to air dry prior to measurement of the mass spectra.

### 13. Deglycosylated α_1_-PI reference

De-pd-α_1_-PI was obtained from pd-α_1_-PI by enzymatic deglycosylation using PNGase F according to the procedure described elsewhere [35]. The de-pd-α_1_-PI was diluted with water to concentration 0.2 μg/μL followed by 1:1 (v/v) dilution with Laemmli buffer, boiled for 3 min and stored (aliquoted) at -20°C until use.

### 14. Non-glycosylated r-α_1_-PI reference produced in *E. coli*

Expression of the human gene for α_1_-PI in *E. coli *special strain BL21(DE3)pLysS (Novagen) was performed as a 9-His-tagged protein as reported earlier [27]. Briefly, the human gene for α_1_-PI was cloned into the pET-19b vector. The expression vector ET-19b, PCR II TOPO vector, TOPO TA Cloning Kit and the restriction enzymes were from (Novagen). After transformation, the host cells Rosetta(DE3)pLysS containing the pET-19b/α_1_-PI construct were grown overnight at 37°C and 250 rpm in LB media containing Ampicillin (100 μg/mL) to a density of 0.8–0.9 OD read at 600 nm (OD_600_). After inoculation (1:100), a culture of LB-medium supplemented with ampicillin (100 μg/mL) was grown (37°C, at 250 rpm) to an OD_600 _of 0.5, and the expression was induced by adding 1.0 mM IPTG. The growth was continued for another 3 h (37°C, 250 rpm) to OD_600 _of 1.1–1.3. The cells were harvested by centrifugation (15 min, 5,000 rpm, 4°C), and washed twice with PBS buffer, pH 7.4, by resuspending and centrifugation. The washed cells were lysed, and the soluble fraction was used for the r-α_1_-PI purification on TALON beads. (Blank culture with pET-19B vector without α_1_-PI gene was performed in a similar manner, and the supernatant served as a control to assure the specificity of α_1_-PI quantification by ELISA and potency measurements.)

## List of abbreviations

α_1_-PI, α_1_- proteinase inhibitor; r-α_1_-PI, recombinant α_1_-PI; *A. niger, Aspergillus niger; *AP, alkaline phosphatase; BApNA, *N*-benzoyl-L-arginine *p*-nitroanalide hydrochloride; de-pd-α_1_-PI, deglycosylated pd-α_1_-PI; ELISA, Enzyme-Linked ImmunoSorbent Assay; glaA, glucoamylase A; HRP, horse radish peroxidase; IPTG, isopropyl β-D-1-thiogalacto-pyranoside; MALDI-MS, matrix-assisted laser desorption/ionization mass spectrometry; NPGB, *p*-nitrophenyl *p*'-guanidino-benzoate hydrochloride; pd-, plasma-derived; RT, room temperature; SDS-PAGE, sodium dodecyl sulphate polyacrylamide gel electrophoresis; SE-HPLC, size-exclusion high-performance liquid chromatography; serpin, serine protease inhibitor; U, uridine.

## Competing interests

The author(s) declare that they have no competing interests.

## Authors' contributions

EK and YO initiated the project. EK performed biochemical and analytical protein characterization and drafted the manuscript. YO did all molecular construction and DNA analysis. LT carried out the transformation and ran the shake-flask growth. ND assisted in initial transformant selection and screening. EK, YO, YS, BG and PP participated in design and coordination of experiments. PP provided strains and molecular tools, and shared his expertise by consulting.

All authors read and approved the final version of the manuscript.
